# Compact Multilayer Yagi-Uda Based Antenna for IoT/5G Sensors

**DOI:** 10.3390/s18092914

**Published:** 2018-09-02

**Authors:** Amélia Ramos, Tiago Varum, João N. Matos

**Affiliations:** 1 Universidade de Aveiro, Campus Universitário de Santiago, 3810-135 Aveiro, Portugal; matos@ua.pt; 2 Instituto de Telecomunicações, Campus Universitário de Santiago, 3810-135 Aveiro, Portugal; tiagovarum@ua.pt

**Keywords:** Yagi-Uda, multilayered antenna, millimeter-waves, IoT

## Abstract

To increase the capacity and performance of communication systems, the new generation of mobile communications (5G) will use frequency bands in the mmWave region, where new challenges arise. These challenges can be partially overcome by using higher gain antennas, Multiple-Input Multiple-Output (MIMO), or beamforming techniques. Yagi-Uda antennas combine high gain with low cost and reduced size, and might result in compact and efficient antennas to be used in Internet of Thins (IoT) sensors. The design of a compact multilayer Yagi for IoT sensors is presented, operating at 24 GHz, and a comparative analysis with a planar printed version is shown. The stacked prototype reveals an improvement of the antenna’s main properties, achieving 10.9 dBi, 2 dBi more than the planar structure. In addition, the multilayer antenna shows larger bandwidth than the planar; 6.9 GHz compared with 4.42 GHz. The analysis conducted acknowledges the huge potential of these stacked structures for IoT applications, as an alternative to planar implementations.

## 1. Introduction

**Figure 1:**
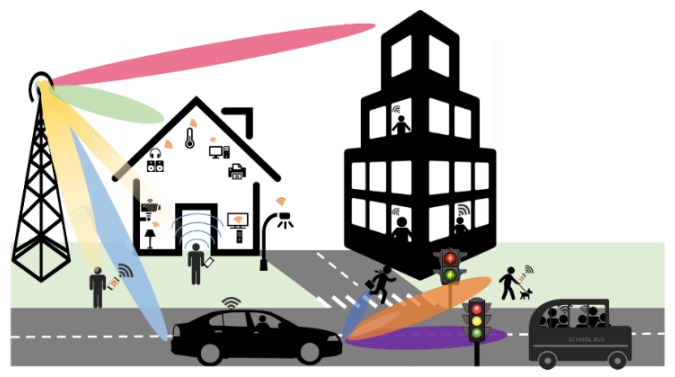
Example of 5G communications and a massive Internet of Things (IoT) scenario.

People’s interconnection was improved by 4G, making the communication more efficient, fluent, and natural. Nowadays, 5G intends to continue that demand and take it to a higher level. In this framework, 5G aims to guarantee people’s interaction, devices interconnection, as well as the connection among people and devices. A network composed of users, daily gadgets, traffic information, personal health conditions, or the status of all the home electronic devices is only a small example of a possible scheme, shown in Figure 1, within many 5G scenarios that are not yet discovered. For now, it is known that 5G services will be divided in three categories; namely, enhanced mobile broadband, mission-critical control, and massive Internet of Things (IoT) [1].

Probably the most effective method to fulfil some demands for 5G cellular services, which are expected to be commercially available in 2020, is to increase the bandwidth [2]; hence, the migration to higher frequencies (in the mmWaves) is mandatory, mainly to support the required gigabit data rate service [3]. On the other hand, as a disadvantage, the frequency ranges of mmWaves present higher path-loss resulting in fragile link, due to weak diffractions at these frequency bands. To overcome these issues, high gain antennas and highly directive antenna arrays using several elements, as well as the application of beamforming techniques, can be used, in order to transmit the wave in the right direction. Yagi-Uda antennas present several characteristics that make them suitable for application in sensors or in larger antenna arrays.

Structurally Yagi-Uda antennas are well known. They are composed of a driven dipole, a reflector element, and a variable number of directors. This scheme guarantees the creation of an end-fire beam [4], appropriate for beamforming applications. This antenna’s layout has been widely analyzed, and historically used, mainly because these structures present a reasonable bandwidth and relatively high gains at moderated costs [5]. With the emergence of printed circuits, several Yagi-like antennas have been proposed for different frequencies of operation and a wide number of applications [6,7].

In Internet of Things (IoT) scenarios, antennas are to be implemented in every small gadget or wearable gadget, mainly to assure the interaction between sensors placed in the most various devices and people. Because of their widespread use, it is highly recommended that antennas are small and compact.

Over the years, some effort has been made to find antenna miniaturization techniques that allow reducing antenna size, maintaining or improving its main characteristics. Some of these methods are described in the literature [8], presented in different classes, including techniques such as fractals, metamaterials, high dielectric constant substrates, slow wave structures, or engineered ground planes. A miniaturization technique described in the literature that is widely used because it also allows obtaining broadband characteristics is the use of reactive impedance substrates (RIS) [9]. In the works of [10,11], two patches are presented, designed on an RIS, which convert them into Ultra-wideband (UWB) antennas. In another work [10], the bandwidth increases over than 100 times, compared with the conventional patch antenna, while in the work of [11], the bandwidth is increased by a factor of 16. These solutions based on RIS are, however, complex to design in millimeter waves, and are used in antenna structures with ground planes.

In addition, for IoT and 5G applications, gain is a concern and even though Yagi antennas naturally present higher gains than microstrip patch antennas [4], this work intends to analyze the advantages of implementing a Yagi-Uda antenna in a multilayer configuration to further enhance its most important properties, such as the gain and the bandwidth.

Multilayer structures have been tested as an attempt to increase the antenna’s gain. In the works of [12,13], two microstrip patch antennas are presented in a multilayer configuration. In the work of [12], an antenna composed of two stacked patches is proposed as a low-cost solution, operating around 3 GHz. The antenna’s bandwidth is 14%, but the information about the radiation pattern and the gain is not provided. On the other hand, in another paper [13], an antenna based on a microstrip patch is presented. It is formed by three parasitic patches working as director elements, operating around 10 GHz. This antenna uses a dielectric foam to ensure the distance between directors, as well as a V-shaped ground plane to improve the reflection coefficient. This antenna has about 190 MHz bandwidth and gain of 11.85 dB, although at the expense of some complexity, and any prototype built is not shown. Additionally, in the work of [14], a structure of eight layers in Low Temperature Co-fired Ceramic (LTCC) technology is presented, also designed for 10 GHz. This antenna consists of a microstrip patch using a proximity coupled feed, presenting a gain of only 3.43 dBi and an S_11_ of −11.52 dB at 10 GHz, in addition to a narrow bandwidth. Moreover, a slot Yagi-like multilayered antenna is proposed by the authors of [15] for 4.2 GHz. This is a vertically stacked structure with three patches, which uses slit slots as the parasitic elements. The prototype shows a bandwidth of about 28% and a gain of 12.20 dBi. However, this structure is bulky and complex. Furthermore, a multilayer substrate integrated waveguide horn antenna array is proposed in another paper [16], in a 4 × 4 array configuration. This antenna uses multilayer cavities with gradually decreased permittivity and expanded aperture sizes above the slots to increase the bandwidth. The array operates between 22.4 to 29.8 GHz (about 28.4% bandwidth) and presents a gain around 15 dBi; however, its complexity is huge.

The authors of [17] designed a Yagi antenna resonant at 5.8 GHz using air gaps, presenting a bandwidth of 14% and a gain of 11 dBi. These air gaps between layers are function of the operation frequency, and this antenna requires a huge spacing between the elements, turning the antenna bulky and likely to vary its physical structure and properties over the time. 

The authors of [18] implemented a multilayer Yagi-like microstrip patch antenna, without air gaps. A gain of 11 dBi was reached and this antenna presents a reasonable measured bandwidth of nearly 20%. However, the structure uses foam between the directors, with low permittivity, which increases the volume of the antenna. No efficiency values are referred to in this work. This antenna is resonant at 10 GHz, which is still a low frequency for the 5G and the massive IoT referred scenarios. A multilayered antenna for 60 GHz is proposed in the work of [19]. This antenna has a reduced size and presents an individual gain of 11 dBi. However, its bandwidth is just 4.2%, which is a clear disadvantage considering the high traffic data rates that, predictably, will be witnessed.

In this paper, two printed antennas are presented for IoT sensors, operating at 24 GHz, and designed according to the theoretical principles of Yagi-Uda structures. A planar printed Yagi-Uda is designed and characterized, and its prototype is shown. This antenna has a gain of 8.9 dBi and a bandwidth of 18%. Then, a compact multilayer stacked antenna is presented. This antenna improves all the main properties of the planar printed Yagi and outperforms the previously referred antennas, showing a gain of 10.9 dBi, an operation bandwidth of 27%, and a total efficiency of 86%, besides its reduced size. This antenna is also an interesting element to be used in larger arrays, and is suited for beamforming applications.

This paper is divided into five sections, starting with an introduction regarding some of the possible applications for these prototypes, where our goals are settled. In the second section, the required features to design the proposed antennas are presented, and in Section 3, the simulation and measured results of both antennas built are shown. Then, the fourth section presents a light discussion on the results obtained, where mainly a comparison between both antennas is highlighted. Lastly, in section five, the main conclusions are reported.

## 2. Materials and Methods

A Yagi-Uda antenna is a directive radiating structure composed of an active element and a group of passive elements, commonly referred to as directors and reflector, with the objective to steer the radiation to a specific direction [5]. Traditionally, Yagi antennas were based on metallic wire structures, in which the directors, reflectors, and the half-wavelength dipole were created [4]. However, and because printed antennas and circuits are currently widely used in microwave applications, several researchers have implemented printed Yagi-like structures in a planar dielectric substrate. Printed antennas allow one to develop low-cost and low-profile antennas, highly versatile and with good efficiency, facilitating its integration in microwave circuits [20]. 

Design concerns of such antenna lie on the estimation of the lengths of the various constituent elements of the antenna, the distance between them, and, possibly, the number of directors needed to accomplish the goals. In this work, the optimal length for the dipole is not equivalent to half-wavelength in vacuum because these antennas are to be printed on a dielectric substrate, and its dielectric constant has a direct impact, causing a reduction on the dipole’s length [21]. In fact, in the literature [21], it was found that the optimal length for a printed dipole would be 0.38 λ. Consequently, an overall reduction on the antenna’s size occurs, which is a clear advantage when considering the target applications for which these antennas can be applied.

When designing antennas for this frequency band, one of the main challenges is the choice of the dielectric substrate to use, as it has a strong impact in the design and in the performance of the antenna. Moreover, at these frequencies, because of the small size of the elements, a more cautious design is required to guarantee an appropriate performance at 24 GHz. In this work, the substrate used is the Rogers RO4350B, because of its good performance in high frequencies (as it presents low dielectric tolerance and loss), as well as its electrical stability properties over frequency. The main characteristics include a relative dielectric permittivity of ε_r_ = 3.48, a dissipation factor of tan(δ) = 0.0037 with a thickness of h = 0.762 mm.

Usually, in Yagi antennas, the spacing between directors varies between 0.1 λ and 0.3 λ and their length is 5% to 30% shorter than the active element [6]. On the other hand, the reflector typically is up to 5% longer than the dipole and it backs the driver [6]. This placement of the active and parasitic elements ensures the end-fire beam formation [4]. In this work, the design of two Yagi antennas is presented, where the first one follows a planar structure and the second is a multilayer assembly of dielectric substrates with the elements printed on them. Both antennas are composed by a dipole, a reflector and three director elements, which is a trade-off between the gain achieved and the overall size of the antennas.

### 2.1. Planar Yagi-Uda Design

The design of the antenna starts with the radiating element, by creating the two arms of the dipole (with global length L_dip_ and width W_dip_) over a substrate layer, which are separated by a small gap, which should be carefully selected as it affects the input impedance of the dipole. Then, three strip directors (with size L_dir_ × W_dir_) were introduced after the dipole, separated by a distance DirSpa, which was adjusted in the simulation process to improve the radiation performance of the antenna. The number of directors was a trade-off between the global size of the antenna and its directivity. 

Then, the feeding part, composed of an impedance matching network, a microstrip balun, and two coplanar printed lines, were connected to the dipole. The coplanar lines allow one to connect each arm of the dipole to each output of the balun, and also to separate the microstrip structure from the dipole of a distance Lcps. This is important because the microstrip section also acts as a reflector element of the Yagi [22]. The balun creates the balanced feeding, by splitting the input signal in two outputs with half-power and half-wavelength phase delay, and finally, a double microstrip transformer matches the input impedance of the antenna to a 50 Ω microstrip line.

**Figure 2:**
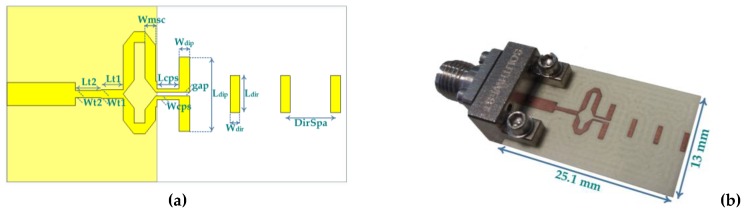
Planar Yagi-Uda antenna: (**a**) layout and parameters; (**b**) photograph of the prototype.

The layout of the designed planar Yagi antenna is presented in Figure 2a. The planar Yagi-Uda antenna was simulated and its dimensions were optimized. After several simulations, the optimal values for the design parameters were accomplished and are summarized in Table 1. As function of the wavelength, it is found that the optimal value for the dipole’s length is equivalent to 0.415 λ, which is reasonably close to theoretical approaches for printed Yagi antennas. The manufactured prototype is shown in Figure 2b. 

### 2.2. Multilayer Yagi-Uda Design

A multilayer version of the printed Yagi antenna was designed with a view to improve the performance and to compact its structure. According to what has been analysed in the literature [23], a gain saturation occurs for planar antennas, especially planar antenna arrays, and developing antennas in a multi-layered arrangement can overcome this limit, because it uses the vertical dimension [19].

The design was based on converting a set of directors and the reflector, properly spaced around the printed dipole. The radiation will be conducted in the upper vertical plane, instead of the horizontal plane, as in the planar version. This radiation characteristic can reveal being a clear advantage for many of the sensors where the antenna will be placed. This type of structure implies that the elements are surrounded in the dielectric substrate. This is one of the differences regarding the planar structure, where the radiation in the antenna follows two different mediums, air and dielectric substrate. Avoiding some air gaps inside the antenna structure keeps it more robust and stable physically, but also in terms of its properties to the environment variations. Thus, in the design process, it is imperative in this application to consider the wavelength in the specified substrate (*λ_d_*).

Considering that the construction of multilayer antennas has a relative novelty, a study on the dielectric and structure was carried out, as a way of better understanding the antenna’s behaviour and the changes produced by structural modifications.

The design of this antenna was based on theoretical dimensions, which were then adjusted through simulation. As in the previous planar form, a dipole was printed on a substrate, and a reflector element using the same line width was placed below at a distance h_ref_. This separation was guaranteed by using a number of layers of dielectric substrate between the elements. 

**Figure 3:**
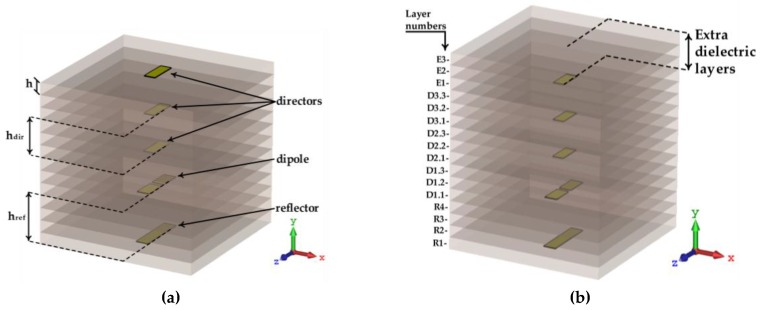
Vertically stacked Yagi antenna: (**a**) without the extra top layers of dielectric; (**b**) with extra layers of substrate.

Several simulations were performed varying the number of directors placed on the structure, with a length of 0.25 *λ_d_*, and spaced by a distance h_dir_. Additionally, the impact of adding a new layer of dielectric material over the last director element was studied. These two versions of structure are shown in Figure 3a,b, respectively. Moreover, Figure 3b highlights the layers identification. Thus, layers *E_n_* constitute the extra dielectric layers (which are to be studied), layers named *D_n.m_*, represent the distance between each director and the following element, meaning the parameter h_dir_, and lastly, *R_n_* layers are representing the distance between the dipole and the reflector. 

**Figure 4:**
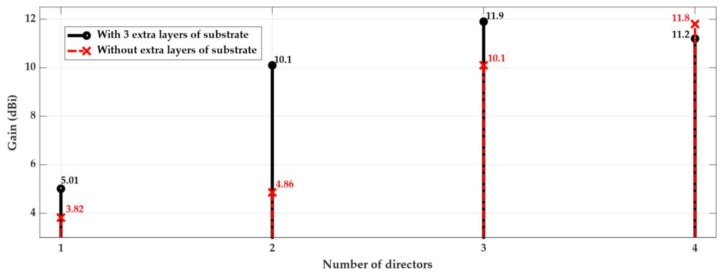
Gain variation with the number of director elements in the Yagi antenna.

**Figure 5:**
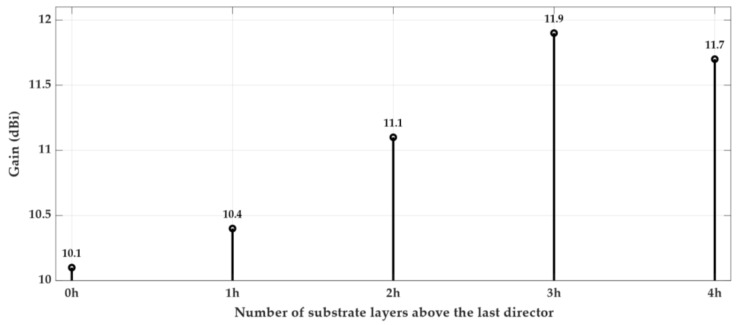
Variation of the gain with the usage of extra layers of substrate topping the last director.

**Figure 6:**
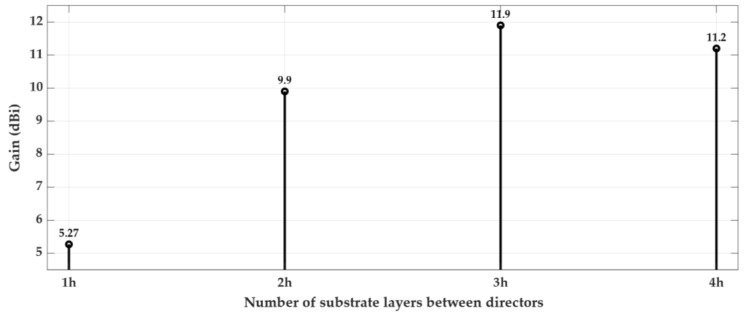
Variation of the gain with the number of dielectric layers between directors.

The analysis was focused mainly on the radiation parameters of the antenna, particularly on its radiation pattern and the obtained gain, because it induces the directivity, which is one of the main parameters that it is required to enhance our application, thus maintaining the compactness of the structure. The results are shown in Figure 4, Figure 5 and Figure 6.

Figure 4 (cross marker) shows the evolution of the antenna’s gain as a function of the number of directors added after the dipole. As expected, the gain increases as the number of directors increases. In parallel, the volume of antenna structure also grows substantially. However, the directors were designed to guide the waves surrounded in dielectric, and the last director has the same length of all the others and is not surrounded as the others are, which led to implementing a slight different scheme.

An alternative design was proposed, as shown in Figure 3b, where on the top of the third director, a few extra layers of dielectric were used. Naturally, this alternative design increases the antenna’s height, but as it is presented in Figure 4 (circle marker), it is clearly compensated by an increase of the antenna’s gain, about 1.8 dBi, using up to three directors. On the other hand, when using four director elements, it is possible to observe a reduction of the gain when the extra dielectric layers are inserted. 

Considering the results of Figure 4, and taking into account the obtained antenna’s gain and its dimensions, using the two possible approaches of Figure 3, three directors were chosen as the optimal compromise for the multilayer Yagi antenna.

Using three directors in the multilayer structure, several simulations were performed mainly to understand the impact of increasing the thickness of the extra substrate layer on the top of the third director. The gain variation due to the number of substrate layers that form the extra dielectric (Figure 3b) is illustrated in Figure 5. The substrate used has a thickness of h = 0.762 mm, and so, the height that tops the last director, as well as the various distances between the other elements, are a multiple of this value.

According to Figure 5, it can be realised that there is a limit to the thickness of the last substrate layer above the last director. The value that maximizes the antenna gain corresponds to 3 h, which is equivalent to 2.286 mm. Finally, to understand the variation of the structure’s gain (using three directors) with the distance between them, in multiples substrate layers (h), a few more simulations were performed, and the results are shown in Figure 6. In this figure, it is verified that the optimal separation between the several directors corresponds to three layers of substrate (3 h), equivalent to h_dir_ = 2.286 mm.

**Figure 7:**
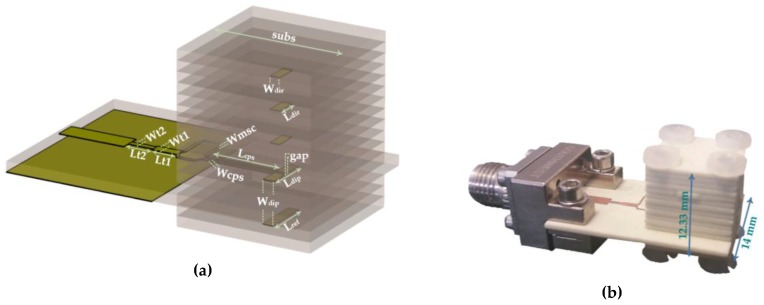
Multilayer Yagi-Uda antenna: **(a)** layout and parameters; **(b)** photograph of the prototype.

Considering the analysis presented above, the multilayer structure of a Yagi-Uda antenna was designed, as is shown in Figure 7a. The multilayer antenna features an optimized compromise between gain and functionality with compact dimensions. It consists of a printed dipole together with three director elements, spaced by a dielectric layer with thickness 3 h, and with a reflector element placed at an optimized distance of: h_ref_ = 4 h. The feeding part of antenna has the same basis of the design used for the planar antenna. Figure 7b shows the prototype of the proposed Yagi-Uda antenna and its final dimensions are mentioned.

After an iterative simulation process, it was possible to achieve the optimal values for the parameters shown in Figure 7a, which are presented in Table 2. Regarding the dipole’s length as a function of *λ_d_*, the optimal length is equivalent to 0.45 *λ_d_*.

## 3. Results

### 3.1. Planar Yagi-Uda: Simulated and Measured

**Figure 8:**
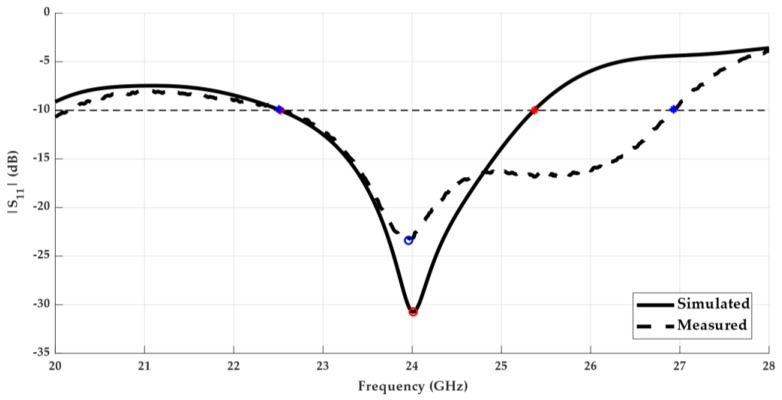
Simulated and measured S_11_ of the designed planar Yagi-Uda antenna.

**Figure 9:**
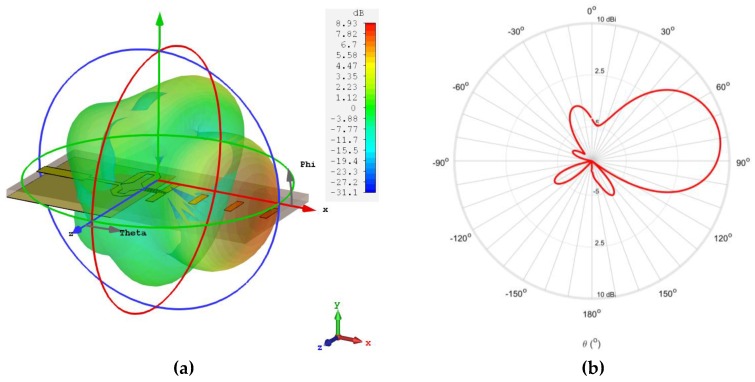
Simulated radiation pattern of the 24 GHz planar antenna: (**a**) 3D view; (**b**) polar diagram of plane φ = 0°.

The planar Yagi antenna depicted in Figure 2 was simulated and characterized in terms of its most important parameters. It was fabricated and measured, and the results are presented in Figure 8 and Figure 9. Figure 8 shows the comparison between the simulated and measured reflection coefficient of the designed antenna. It is possible to observe that the antenna has a good impedance matching at 24 GHz, with a simulated S_11_ of −31 dB and a measured value of −23 dB. 

In terms of operation bandwidth, this antenna presents considerable good results, where the prototype achieved a band of 4.42 GHz [22.5–26.9 GHz] in which it is properly matched, considering the S_11_ < −10 dB criteria, representing a bandwidth of 18%. Comparing these results with the simulated ones, an improvement in the bandwidth was obtained. 

Regarding the radiation properties of the antenna, Figure 9 presents the simulated radiation pattern of the designed antenna, in terms of its 3D view (Figure 9a), and the plane φ = 0° in the 2D polar diagram (Figure 9b). A directive radiation pattern in the horizontal plane, the plane where the directors are placed is observed, with a simulated gain of 8.9 dBi at 24 GHz. The planar antenna also presents a total efficiency of 90%.

### 3.2. Multilayer Yagi-Uda: Simulated and Measured

**Figure 10:**
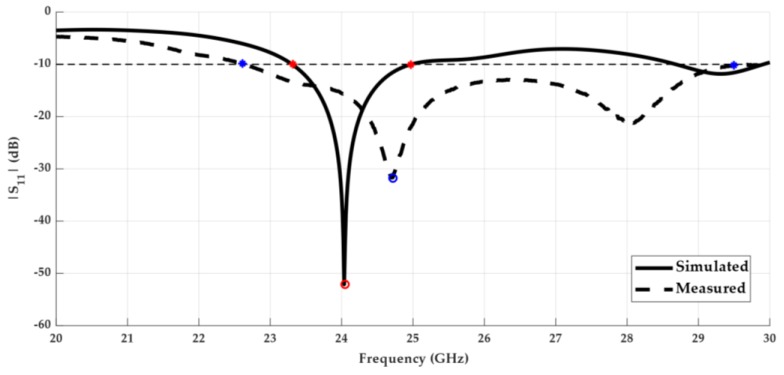
Simulated and measured reflection coefficient of the multilayer Yagi antenna.

Concerning the multilayer antenna from Figure 7, it was simulated and analysed according to its main properties. Figure 10 shows the comparison between the simulated and measured results of the reflection coefficient of the antenna.

A good impedance matching can be observed, showing that the antenna was perfectly tuned in terms of simulation despite the frequency shift of 710 MHz in relation to the measured curve. However, at the operation frequency, 24 GHz, a measured S_11_ of −15.51 dB makes these results satisfactory. Moreover, the designed multilayer antenna presents roughly 6.9 GHz of bandwidth, equivalent to 27%, from 22.6 GHz up to 29.5 GHz—a frankly encouraging result.

**Figure 11:**
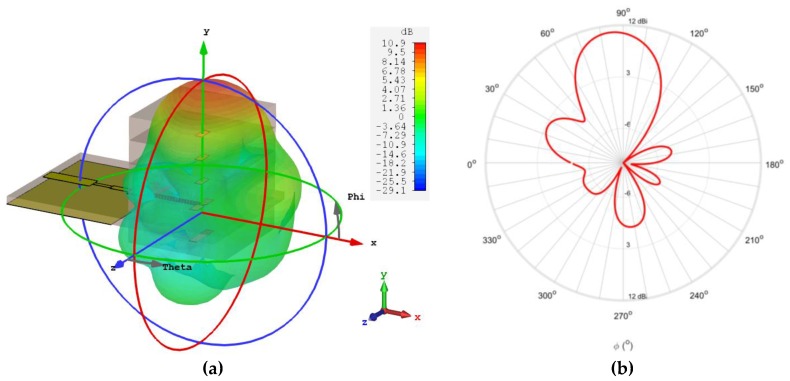
Simulated radiation pattern of the 24 GHz multilayer antenna: (**a**) 3D view; (**b**) polar diagram.

Looking at the radiation pattern, the simulated results are shown in Figure 11, where it is possible to verify the alignment of the main lobe with the directors of the antenna, in accordance with the vertical plane.

**Figure 12:**
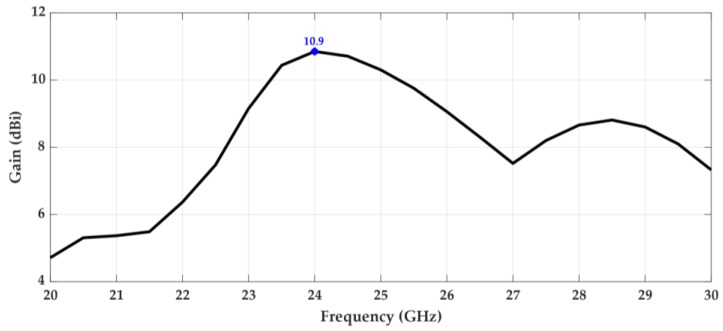
Gain over frequency.

**Figure 13:**
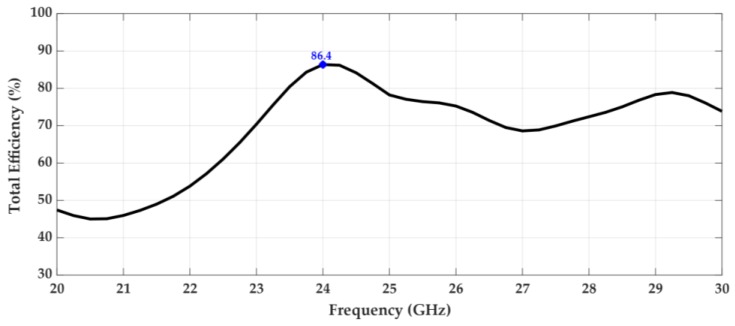
Total efficiency over frequency.

Figure 12 and Figure 13 show the variation of the maximum gain of the antenna and its total efficiency, respectively, over the frequency. It can be seen that the structure has a maximum gain of 10.9 dBi at 24 GHz, the frequency for which it was designed. Furthermore, there is a range of about 2 GHz around the operating frequency where the gain is always greater than 10 dBi. 

In terms of the antenna’s efficiency, according to Figure 13, it is possible to see that it is high, about 86% at the 24 GHz frequency. This result is very important, given the complexity of the structure, its compactness, consisting of several stacked substrate dielectric layers. It should also be noted that between 23.5 and 24.9 GHz, the total efficiency of the antenna is always greater than 80%.

## 4. Discussions

Regarding the feeding structure, it was verified that the used microstrip-to-coplanar stripline transition provides good matching results between the simulated and practical measurements, for both designed antennas. However, this microstrip structure is not physically symmetrical, because of the balun’s arms that cause the 180° phase shift. This asymmetry causes a slight deviation on the main lobe of the radiation pattern in the planar antenna. In the multilayer implementation, the influence of the balun in radiation is negligible, as a reflector element was used, and the main radiation lobe is vertically aligned with the directors.

Furthermore, the impedance matching made on the planar antenna is very accurate, however, in the multilayer antenna, a small frequency shift was observed. This might be a consequence of the construction method of this antenna, which was very simple, just to demonstrate the concept. In fact, this vertically stacked Yagi-Uda antenna has its elements surrounded in substrate, and, possibly, there may have existed a slight difference between the practical and theoretical, used in simulation, and as a result, the minimum return loss happens at a slight different frequency than in the simulation.

The concept of converting the planar into a multilayer structure, as shown in Table 3, is clearly promising, as not only it is possible to reduce the antenna’s overall size, but also, improvements are seen, both in bandwidth and gain.

## 5. Conclusions

In this paper, two compact printed antennas, based on Yagi structures, were presented as a solution to integrate IoT sensors. A multilayer antenna, which is an adaptation of the conventional planar Yagi structure was designed, improving the most important properties compared with the planar antenna and with the state-of-the-art. The multilayer antenna designed provides more than 25% bandwidth, covering the entire 5G allocated band in Europe, allowing its usage for IoT applications. Its improved gain to almost 11 dBi in a cube structure of nearly 1 cm^3^ of volume is a good achievement to combat the propagation issues of use mmWave frequencies, a gain considerably higher than the conventional microstrip patch antennas. 

Moreover, the multilayer configuration permits to change the direction of radiation to the vertical plane, allowing it to be placed over the IoT sensors circuits. These results were not obtained at the expense of a reduction of antenna efficiency, because the multilayer structure shows an efficiency of 86%. Finally, the multilayer antenna has modular physical features that allow it to be easily grouped into high gain antenna arrays, or also arrays for 5G applications using beamforming techniques. Thus, the multilayer antenna presents better radiation performance, essentially, better gain and a wider bandwidth while being simultaneously smaller and more compact.

In conclusion, the possibility of using both Yagi antennas in 5G scenarios, physical sensors, or as a singular element of a larger array has been confirmed. Moreover, a multilayer implementation seems to be a promising chance for achieving better performances at higher frequencies.

## Figures and Tables

**Table 1:** Parameters of the 24 GHz planar Yagi-Uda antenna (UNITS: mm).

L_dip_	L_dir_	W_dip_	W_dir_	gap	DirSpa	L_cps_	W_cps_	W_msc_	L_t1_	L_t2_	W_t1_	W_t2_
5.19	2.75	0.8	0.72	0.3	3	1.63	0.25	0.8	1.64	1.91	0.65	0.54

**Table 2:** Parameters of the 24 GHz multilayer Yagi-Uda antenna (UNITS: mm).

**Subtable 2.1. Lengths of the lines used.**					
**L_dip_**	**L_dir_**	**L_ref_**	**L_cps_**	**L_t1_**	**L_t2_**
3	1.68	3.47	5.32	1.88	1.918
**Subtable 2.2. Widths of the lines used.**					
**W_dip_**	**W_dir_**	**W_cps_**	**W_msc_**	**W_t1_**	**W_t2_**
0.80	0.715	0.25	0.25	0.78	0.48
**Subtable 2.3. Other parameters of design**.					
**h_dir_**		**h_ref_**	**subs**		**gap**
2.286		3.048	10		0.3

**Table 3:** Measured bandwidth and simulated gain of both antennas.

Antenna	Bandwidth	Gain
Planar	4.42 GHz 18%	8.9 dBi
Multilayer	6.9 GHz 27%	10.9 dBi
